# Microglia-neuron communication in ischemic stroke: from homeostatic signaling to pathological remodeling and therapeutic targeting

**DOI:** 10.3389/fmolb.2026.1910699

**Published:** 2026-09-03

**Authors:** Sijie Liu, Hao Huang, Jian Xiong, Shuhong Yu, Yi Luo, Biao Zhang

**Affiliations:** 1 Department of Neurology, Suzhou Hospital of Integrated Traditional Chinese and Western Medicine, Suzhou, Jiangsu, China; 2 Institute of Public Health, Suzhou Vocational Health College, Suzhou, Jiangsu, China; 3 Central Laboratory, Suzhou Hospital of Integrated Traditional Chinese and Western Medicine, Suzhou, Jiangsu, China

**Keywords:** extracellular vesicles, ischemic stroke, microglia, neuroinflammation, neuron, phagocytosis, synaptic remodeling

## Abstract

Ischemic stroke profoundly disrupts the homeostatic dialogue between microglia and neurons, converting a physiological surveillance network into a dynamic, injury-responsive, and spatially heterogeneous communication system. Under physiological conditions, neurons regulate microglia through diverse signaling pathways, while microglia support neuronal health via synaptic remodeling, inflammatory regulation, metabolic balance, and debris clearance. Following cerebral ischemia, cascades involving excitotoxicity, energy failure, ionic imbalance, damage-associated molecular patterns (DAMPs), complement activation, and cell death signals rapidly reprogram this bidirectional communication. In this Review, we propose the Microglia-Neuron Communication Continuum (MNCC) model as a conceptual framework for understanding post-stroke microglia-neuron crosstalk. The MNCC model conceptualizes these interactions as a multidimensional continuum defined by three core axes: time, communication mode, and functional outcome. Along the temporal axis, crosstalk evolves from the hyperacute and acute phases to the subacute and chronic stages. Along the communication-mode axis, interactions span direct contact-dependent mechanisms and indirect soluble signaling pathways. Along the functional-output axis, the biological consequences range from damage amplification to tissue repair and chronic maladaptation. Using this framework, we first summarize the physiological basis of bidirectional microglia-neuron signaling under homeostatic conditions. We then examine how ischemic stroke reprograms this network across distinct temporal stages and spatial niches, emphasizing that post-stroke crosstalk is a continuous, context-dependent, spatially heterogeneous, and functionally plastic process. Finally, we discuss current and emerging therapeutic strategies through the lens of the MNCC model, focusing on temporal matching, dominant communication modes, regional heterogeneity, and translational barriers. A deeper understanding of this communication continuum may facilitate the development of targeted interventions that restrain harmful signaling, preserve beneficial interactions, and ultimately improve long-term neurological recovery after ischemic stroke.

## 1 Introduction

Ischemic stroke remains a leading cause of mortality and long-term disability worldwide (Palaniappan et al., 2026; Liu et al., 2025). The abrupt interruption of cerebral blood flow initiates a complex pathogenic cascade characterized by excitotoxicity, oxidative stress, ionic imbalance, mitochondrial dysfunction, blood-brain barrier (BBB) disruption, and robust neuroinflammation. For decades, experimental research focused on preventing neuronal death by directly targeting excitotoxicity, oxidative stress, and calcium overload. Despite a robust preclinical rationale, most of these neuroprotective strategies have failed to yield clinical benefits (Tauskela and Blondeau, 2025; Wang M. et al., 2025). These setbacks strongly suggest that neuronal fate after ischemia is not cell-autonomous. Although neurons bear the brunt of ischemic injury, the ultimate trajectory of tissue damage and repair is driven by reciprocal crosstalk among various cell types within the post-ischemic microenvironment. Among these cellular networks, communication between microglia and neurons plays a central role (Tiedt et al., 2022).

Microglia constitute the primary resident immune population within the central nervous system (CNS). Rather than acting as passive observers, they serve as active sentinels that constantly sample the local environment and promptly reprogram their functional phenotypes upon ischemic insult (Qiao et al., 2023; Qin et al., 2019). In turn, neurons are not merely passive victims of injury, they provide instructive signals that restrain excessive microglial activation and guide microglial surveillance (Lénárt et al., 2024). This bidirectional relationship enables rapid adaptation to alterations in neuronal activity and tissue status. Following an ischemic stroke, however, this homeostatic dialogue is profoundly disrupted. Depending on the temporal stage, anatomical location, severity of injury, and cellular metabolic state, microglia-neuron communication can either amplify neuronal damage or support tissue repair (Yu et al., 2022).

Historically, post-stroke microglial responses were often interpreted using simplified frameworks, such as “resting *versus* activated” states or the binary M1/M2 polarization paradigm (Wang et al., 2023). While these concepts have been valuable heuristic tools, they are insufficient to describe the remarkable diversity and plasticity of microglial states revealed by modern single-cell transcriptomic, spatial omics, and *in vivo* imaging studies (Tian et al., 2026). Accumulating evidence indicates that the same molecular pathway may exert divergent or even opposing effects depending on the timing, local microenvironment, neuronal viability, and phenotypic context (Han B. et al., 2024).

To integrate these complex, overlapping processes, we propose the Microglia-Neuron Communication Continuum (MNCC) model. This model conceptualizes post-stroke microglia-neuron crosstalk as a multidimensional continuum rather than a linear cascade or a simple binary switch. The MNCC model comprises three core axes: the temporal axis, the communication-mode axis, and the functional-output axis. In addition to these three dimensions, spatial heterogeneity and cellular state act as critical modifiers. Consequently, the MNCC model emphasizes that effective therapeutic strategies should not aim to globally suppress microglia or inflammation. Instead, they should be designed to reshape aberrant cellular communication while preserving or enhancing reparative signals in a time- and region-specific manner.

In this Review, we first outline the physiological basis of microglia-neuron communication under homeostatic conditions. We then discuss how ischemic stroke disrupts and reprograms this reciprocal signaling network across temporal and spatial dimensions, focusing on inflammatory mediators, phagocytic checkpoints, metabolic coupling, extracellular vesicle signaling, and organelle transfer. Finally, we examine current and emerging therapeutic strategies targeting this communication axis and discuss the major challenges that must be overcome to successfully translate these mechanistic insights into clinical interventions.

## 2 Physiological basis of microglia-neuron signaling crosstalk

### 2.1 Neuron-derived “calming signals” that restrain microglial activation

Under physiological conditions, neurons provide multiple constitutive signals that instruct microglial behavior and maintain them in a homeostatic state (Zhao et al., 2024). These regulatory signals include the CX3CL1-CX3CR1 axis, the CD200-CD200R axis, neuronal ATP/ADP-mediated purinergic signaling, and various membrane-bound or soluble molecules that modulate microglial inflammatory thresholds (Pawelec et al., 2020; Zhao et al., 2019; Illes et al., 2020). Through these mechanisms, neurons actively communicate their functional status to neighboring microglia, thereby preventing inappropriate inflammatory activation while preserving the capacity for rapid immune responsiveness.

The CX3CL1-CX3CR1 pathway is one of the best-characterized neuron-to-microglia communication systems. CX3CL1 is highly expressed by neurons, whereas its cognate receptor, CX3CR1, is predominantly restricted to microglia within the CNS. Under homeostatic conditions, this axis restrains excessive microglial activation, regulates synaptic maturation, and contributes to neuronal survival (He et al., 2025). Disruption of CX3CL1-CX3CR1 signaling alters microglial reactivity and impairs synaptic plasticity, highlighting its indispensability for maintaining balanced microglia-neuron communication (Pawelec et al., 2020).

The CD200-CD200R pathway represents another critical inhibitory checkpoint. CD200 is expressed by neurons and other brain cells, whereas CD200R is expressed by microglia. Engagement of this pathway suppresses microglial inflammatory responses and maintains immune quiescence. Under conditions of neuronal stress or injury, reduced CD200 expression may remove this inhibitory signal, allowing microglia to shift toward a reactive state (Zhao et al., 2019).

Beyond these immune-checkpoint pathways, neuronal activity directly influences microglia through neurotransmitters and neuromodulators. Microglia express a wide spectrum of receptors for glutamate, gamma-aminobutyric acid (GABA), acetylcholine, norepinephrine, dopamine, and adenosine, enabling them to sense neuronal firing patterns and network activity (Albertini et al., 2020). Through these diverse neurotransmitter systems, the functional state of neuronal circuits directly shapes microglial surveillance, migration, inflammation, and synaptic regulation. Collectively, these pathways demonstrate a central principle: by imposing inhibitory constraints via chemokine, cell-surface, and neurotransmitter signaling, neurons actively define the activation threshold, surveillance profile, and effector limits of microglia under physiological conditions.

### 2.2 Microglial “surveillance and stewardship” that preserve neuronal homeostasis and circuit stability

In the healthy CNS, microglia serve as highly dynamic sentinels that continuously monitor the parenchymal microenvironment. Ramified microglia constantly extend and retract their highly motile processes to survey the local milieu, contacting synapses, axons, dendrites, and neuronal somata in an activity-dependent manner (Petry et al., 2023) (Table 1). Purinergic signaling is central to this baseline surveillance. Neuronal activity and local tissue stress tightly regulate extracellular ATP and ADP levels, which are sensed by microglial purinergic receptors (Illes et al., 2020). Following ischemia, excessive ATP release and altered purinergic receptor engagement drive microglial migration toward injured regions and amplify inflammatory responses. This high sensitivity allows microglia to serve as early responders to microenvironmental imbalances before overt structural damage occurs (Cserép et al., 2020). Hence, dynamic surveillance constitutes the primary layer of crosstalk between microglia and neurons, laying the groundwork for either protective or pathological responses after ischemic injury.

In addition to immune surveillance, microglia actively contribute to synaptic remodeling throughout development and adulthood. They participate in activity-dependent synaptic pruning, monitor synaptic integrity, and modulate neurotransmission through both direct contact and soluble mediators (Stevens and Johnson, 2021). Complement components, particularly C1q and C3, tag synapses destined for elimination, whereas microglial complement receptor 3 (CR3) mediates synaptic engulfment (Stevens et al., 2007). While this mechanism is essential for developmental circuit refinement, its aberrant reactivation after injury can contribute to pathological synapse loss (Han Q. Q. et al., 2024). Microglia also influence synaptic function by releasing cytokines, growth factors, lipid mediators, and neurotrophic molecules. Signaling through molecules such as transforming growth factor-beta (TGF-β), insulin-like growth factor 1 (IGF-1), and brain-derived neurotrophic factor (BDNF) can modulate synaptic strength and plasticity (Wiens et al., 2024).

To maintain a healthy brain microenvironment, microglia express various ion channels and transporters, including Na^+^/H^+^ exchanger 1 and Na^+^/Ca^2+^ exchanger (Verkhratsky et al., 2021). These proteins help stabilize extracellular ion concentrations, regulate pH, and maintain Ca^2+^ balance, thereby supporting baseline neuronal excitability and synaptic transmission (Luo et al., 2021). In parallel, microglia express multiple phagocytic receptors that enable the efficient engulfment of apoptotic cells, myelin debris, and harmful extracellular aggregates (Butler et al., 2021). Together, these homeostatic stewardship activities protect neurons from toxic damage and maintain tissue integrity.

**FIGURE 1:**
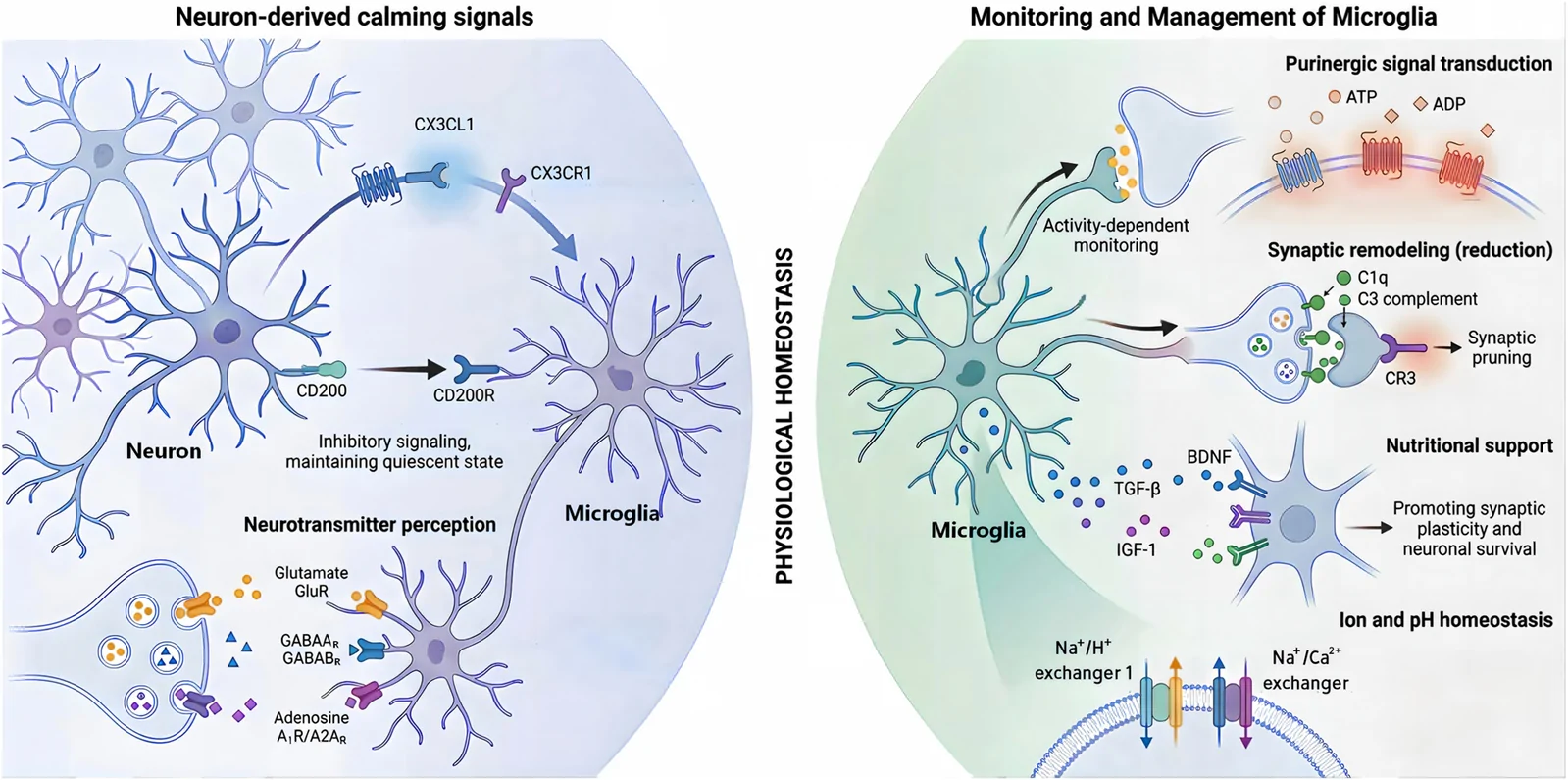
Bidirectional microglia-neuron signaling crosstalk under physiological homeostasis. Under healthy conditions, the central nervous system maintains structural and functional stability through a dynamic equilibrium between neurons and microglia. The left panel illustrates neuron-derived constitutive “calming signals” that restrain microglial overactivation, primarily mediated by the CX3CL1-CX3CR1 and CD200-CD200R immune checkpoints, alongside microglial neurotransmitter receptors that continuously monitor network activity. The right panel depicts microglial “surveillance and stewardship” mechanisms that preserve neuronal homeostasis. Guided by purinergic signal transduction, ramified microglia constantly sample the local environment. They regulate neural circuit stability via activity-dependent synaptic pruning, provide critical nutritional support via neurotrophic factors, and stabilize extracellular excitability through ion and pH homeostatic regulators.

Thus, under physiological conditions, communication between microglia and neurons is best characterized as a bidirectional, dynamic equilibrium (Marinelli et al., 2019). Even in the absence of overt injury, both neuronal instructive signals and microglial response states are continuously recalibrated according to developmental stage, brain region, neuronal activity, metabolic status, and systemic context. The physiological logic of this crosstalk provides the essential framework for understanding how this relationship becomes disrupted after an ischemic stroke. The very pathways that preserve homeostasis are precisely those that, when dysregulated under pathological conditions, contribute to neuroinflammation, neuronal injury, and functional impairment. Recognizing this continuity between homeostatic and pathological states is critical for developing therapeutic strategies aimed not at broadly suppressing microglial function, but at restoring balanced, bidirectional communication (Figure 1).

**TABLE 1:** Temporal organization of microglia-neuron communication after ischemic stroke.

Stage	Approximate time window	Neuronal status	Microglial response	Functional consequences	References
Hyperacute	Minutes to hours	Energy failure, membrane depolarization, excitotoxic glutamate release, and robust release of DAMPs	Rapid sensing of distress signals, directional process extension via purinergic receptors	Early injury sensing, potential inflammatory priming	Majumder, 2024; Alonso-Alonso et al., 2022; Sakai and Shichita, 2023; Fan et al., 2017; Cserép et al., 2020
Acute	Hours to 3 days	Apoptosis, necrosis, oxidative stress, complement tagging	Morphological transformation into an amoeboid phenotype; robust release of pro-inflammatory mediators; initiation of synaptic phagocytosis	Neurotoxicity, damage amplification, or containment of irreversible focal tissue injury	Dirnagl et al., 1999; Zong et al., 2026; Qiao et al., 2023; Jia et al., 2022; Liu et al., 2020; Yu et al., 2025; Chen et al., 2022; Brown, 2021
Subacute	3–14 days	Partial survival in the penumbra, persistent synaptic instability, and initiation of early structural tissue remodeling	Phenotypic shifting toward an anti-inflammatory state; enhanced efferocytosis and debris clearance; secretion of neurotrophic factors	Active resolution of inflammation, tissue remodeling, or maladaptive synaptic pruning	Fan et al., 2023; Kwon and Koh, 2020; Chai et al., 2022; Rajkovic et al., 2018; Schafer et al., 2012; Dundee et al., 2023; Planas, 2024
Chronic	Weeks to months	Neural circuit reorganization, axonal sprouting, dendritic spine remodeling, or delayed progressive neuronal loss	Persistent low-grade activation or specialized remodeling states	Functional neurological recovery, adaptive circuit refinement, or chronic sequelae	Yoon et al., 2022; Ball et al., 2022; Qiao et al., 2023; He et al., 2025; Liu et al., 2024; Alawieh et al., 2020

## 3 The MNCC model: a multidimensional framework for post-stroke communication

The MNCC model provides a conceptual structure to organize the complex landscape of microglia-neuron crosstalk after ischemic stroke. Rather than categorizing microglia as strictly harmful or protective, this model positions each communication event within a continuum defined by time, communication mechanism, and functional consequence.

### 3.1 X-axis: temporal organization of communication

Microglia-neuron communication after stroke is strictly dependent on the temporal phase of the disease. In the hyperacute phase, the dominant communication mode within the MNCC model is danger signal-mediated microglial sensing. Neurons rapidly undergo energy failure due to the abrupt cessation of cerebral blood flow, triggering plasma membrane depolarization, excitotoxic glutamate release, intracellular calcium overload, and mitochondrial dysfunction (Majumder, 2024; Alonso-Alonso et al., 2022). These early ischemic events generate a barrage of rapid distress signals collectively termed DAMPs, which include adenosine triphosphate (ATP), glutamate, reactive oxygen species (ROS), mitochondrial DNA (mtDNA), and high-mobility group box 1 (HMGB1) (Sakai and Shichita, 2023).

As the resident immune sentinels of the CNS, microglia are exquisitely sensitive to these early danger signals and respond within minutes. In the acute phase, microglia undergo pronounced morphological and functional transformations, characterized by process retraction, somatic enlargement, migration toward the lesion, and the acquisition of an amoeboid phenotype (Fan et al., 2017; Kaushal and Schlichter, 2008). Fully activated microglia release a battery of pro-inflammatory mediators, including tumor necrosis factor-alpha (TNF-α), IL-1β, interleukin-6 (IL-6), chemokines, nitric oxide, and ROS (Dirnagl et al., 1999). These mediators exacerbate neuronal injury by worsening excitotoxicity, mitochondrial dysfunction, oxidative stress, BBB disruption, and peripheral leukocyte recruitment. Concurrently, the loss or dysfunction of neuronal activity alters the local neurotransmitter milieu, further reshaping microglial inflammatory tone (Zong et al., 2026). Thus, the acute inflammatory response is not a unidirectional process driven solely by microglia.

Another notable feature during this period is the initiation of microglial phagocytosis. Microglia begin to clear dead neurons, fragmented neurites, damaged synapses, myelin debris, and toxic extracellular material (Qiao et al., 2023). This recognition and engulfment are typically mediated by the complement system and specialized phagocytic receptors, including TREM2, CD36, and MerTK (Jia et al., 2022; Liu et al., 2020; Yu et al., 2025). While this process is essential for preventing secondary necrosis and limiting inflammatory propagation (Chen et al., 2022), reactive microglia may inadvertently phagocytose stressed-but-viable neurons in the penumbral region, thereby expanding the zone of irreversible injury (Brown, 2021).

The subacute phase marks a pivotal transition from injury propagation to tissue repair (Fan et al., 2023). This phase involves three interdependent processes: the active resolution of inflammation, the efficient clearance of cellular debris, and the initiation of early structural repair. It represents a transitional MNCC state where destructive and restorative processes coexist. Microglia gradually transition from a pro-inflammatory to an anti-inflammatory phenotype, producing anti-inflammatory cytokines and trophic factors including IL-10, transforming growth factor-β (TGF-β), insulin-like growth factor 1 (IGF-1), and brain-derived neurotrophic factor (BDNF) (Kwon and Koh, 2020). These factors promote neuronal survival, neurite outgrowth, and synaptic plasticity (Chai et al., 2022). Neurotrophic factors secreted by these reparative microglia serve as key drivers of neural remodeling (Rajkovic et al., 2018]. At a finer structural level, microglia actively participate in synaptic remodeling and repair. They precisely identify and eliminate irreparably damaged protrusions and neuronal fragments through the complement system and phagocytic receptors while preserving salvageable structures (Schafer et al., 2012; Dundee et al., 2023). However, if pro-inflammatory signaling remains dominant, microglia can perpetuate inflammation, thereby delaying resolution and impairing tissue repair (Planas, 2024). Furthermore, excessive or misdirected phagocytic activity may remove functionally recoverable synapses, potentially hindering circuit reorganization.

**FIGURE 2:**
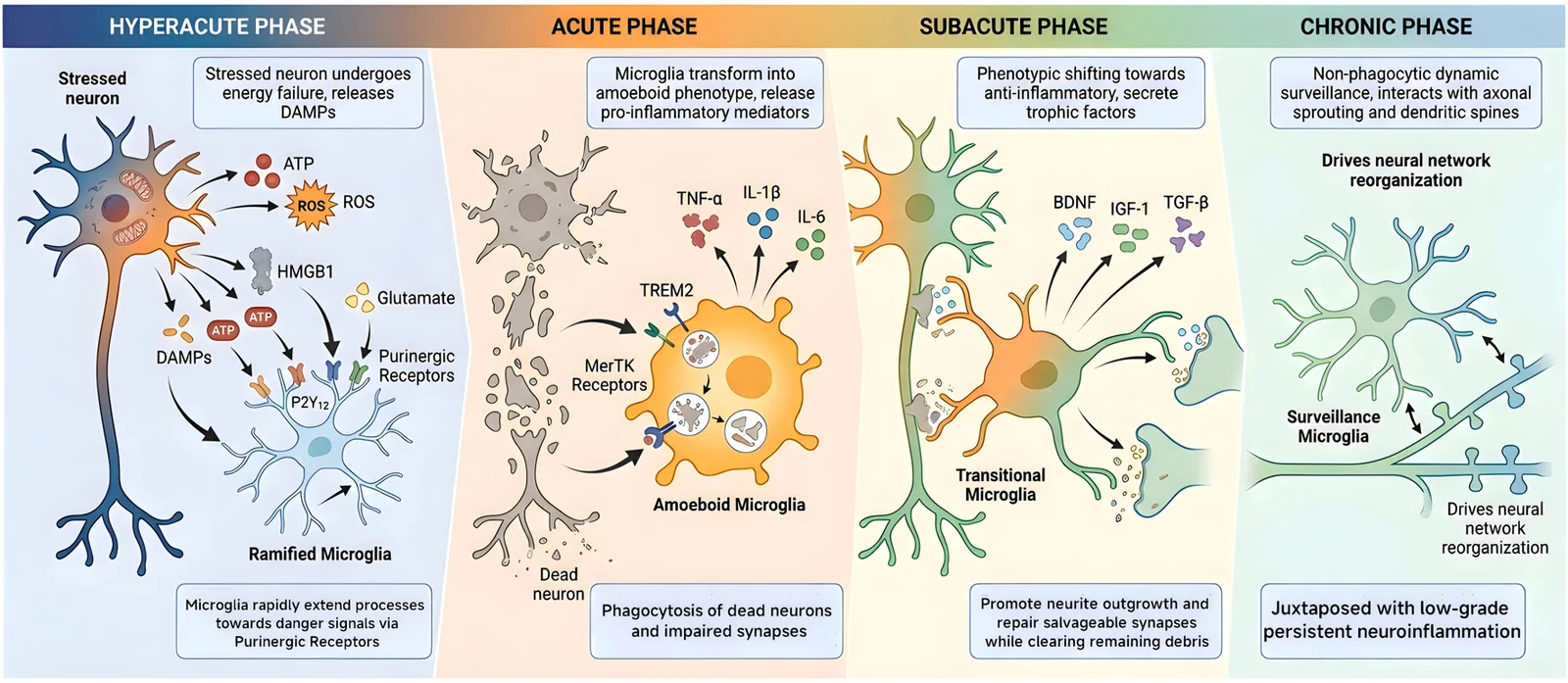
Schematic representation of the MNCC model across post-stroke stages. Following ischemic stroke, the homeostatic dialogue undergoes temporal and functional reprogramming, evolving through four continuous phases. In the Hyperacute Phase, abrupt energy failure causes stressed neurons to release damage-associated molecular patterns, prompting ramified microglia to rapidly extend processes toward the lesion via purinergic signaling. In the Acute Phase, activated microglia transform into an amoeboid phenotype, releasing robust pro-inflammatory mediators that amplify secondary injury, while initiating the phagocytosis of dead neurons and impaired synapses. During the Subacute Phase, a pivotal transition toward tissue repair occurs; transitional microglia adopt an anti-inflammatory state, secreting trophic factors to promote neurite outgrowth and stabilize salvageable synapses while clearing remaining debris. In the Chronic Phase, the microenvironment is characterized by a balance between neural network reorganization and low-grade, persistent neuroinflammation.

In the chronic phase, microglia-neuron communication does not simply return to the pre-stroke homeostatic baseline. Instead, many brain regions exhibit persistent low-grade neuroinflammation, altered synaptic connectivity, long-term metabolic disturbances, and incomplete structural repair (Yoon et al., 2022). Microglia actively participate in synaptic pruning and remodeling through both phagocytic and non-phagocytic mechanisms, shaping the formation of new functional connections (Ball et al., 2022). Concurrently, microglia-derived trophic factors support dendritic spine remodeling, axonal plasticity, and activity-dependent recovery (Qiao et al., 2023). Neurons, in turn, modulate microglial behavior through restored neural activity, neurotransmitter release, chemokine signaling, and membrane-bound regulatory ligands (He et al., 2025). This dynamic interplay drives post-stroke neural circuit reorganization (Qiao et al., 2023). However, persistent, aberrant microglial activation can sustain chronic neuroinflammation, driving delayed neuronal loss and maladaptive plasticity that contribute to long-term neurological sequelae (Liu et al., 2024; Alawieh et al., 2020). In summary, chronic microglia-neuron communication represents a context-dependent balance between adaptive circuit refinement and destructive synaptic loss (Figure 2).

### 3.2 Y-axis: communication modes

The second dimension of the MNCC model delineates communication modes, which form the molecular basis of bidirectional crosstalk under both physiological and ischemic stroke conditions. This communication is mediated by several overlapping, synergistic mechanisms summarized as follows.

Direct contact-dependent interactions rely on membrane ligand-receptor pairs and physical cell-cell junctions for localized signaling. Homeostatic axes, such as CX3CL1-CX3CR1 and CD200-CD200R, are severely disrupted post-stroke, removing vital inhibitory restraints on microglial overactivation (Pawelec et al., 2020; Zhao et al., 2019). Contact-dependent interactions also encompass “eat-me” and “don’t-eat-me” signaling pathways. While the CD47-SIRPα “don’t-eat-me” signal protects stressed but viable neurons, complement fragments deposit onto impaired synapses and bind to microglial complement receptors, triggering pathological synaptic engulfment and persistent synapse loss (Lehrman et al., 2018; Dalakas et al., 2020). Additionally, tunneling nanotubes (TNTs) establish physical cytoplasmic bridges that facilitate direct intercellular material exchange. Emerging evidence indicates that TNTs mediate the direct transfer of mitochondria between cells; in the context of ischemic stroke, this mechanism may allow microglia to clear damaged neuronal mitochondria or provide direct bioenergetic support to stressed neurons (Scheiblich et al., 2024; Piao et al., 2026).

Indirect soluble signaling mediates long-range paracrine inflammatory cascades via diffusible mediators. Hypoxic neurons release ATP through pannexin-1 hemichannels, stimulating microglial chemotaxis and NLRP3 inflammasome activation (Suzuki et al., 2020). Activated microglia secrete pro-inflammatory cytokines and chemokines that aggravate neuronal excitotoxicity, whereas anti-inflammatory cytokines like TGF-β and IL-10 mitigate reperfusion injury during the subacute stage. Other soluble mediators, including excess glutamate, prostaglandins, ROS, and various DAMPs, continuously fuel neuroinflammation within ischemic lesions (Carinci et al., 2021; Fisher and Savitz, 2022). Extracellular vesicles (EVs) are also a means of indirect communication between microglia and neurons. Both neurons and microglia release abundant exosomes and microvesicles post-stroke, carrying miRNAs, inflammatory proteins, lipids, and fragmented mitochondria. Stressed neuron-derived vesicles can prime microglial inflammatory activation (Paolicelli et al., 2019). Accordingly, pro-inflammatory microglia-derived vesicles promote neuronal apoptosis, whereas anti-inflammatory microglia-derived vesicles alleviate excitotoxic injury and facilitate tissue repair (Zhou T. et al., 2025).

Metabolic coupling and remodeling acts as another core module governing the inflammation-to-repair transition within the MNCC model. Under ischemic, energy-deficient conditions, metabolic coupling mechanisms sustain the bidirectional exchange of nutrients and stress signals. Glycolytic microglia produce lactate, which can either fuel neuronal respiration at moderate levels or trigger local acidosis and apoptosis at high concentrations (Li et al., 2025). Furthermore, dysregulated lipid metabolites, mitochondrial ROS, and NAD^+^ depletion jointly reshape microglial immunometabolism, driving pro-inflammatory polarization and propagating neuronal oxidative damage (Wei et al., 2024; Wang M. et al., 2025).

Importantly, these communication modes do not operate in isolation within the ischemic brain; instead, they establish intricate cross-regulatory networks that undergo dynamic alterations as the stroke progresses. For instance, neuronal ATP release simultaneously induces microglial migration toward injured synapses and activates the microglial NLRP3 inflammasome (Badimon et al., 2020; Suzuki et al., 2020]. Complement labeling acts in concert with specific phagocytic receptors to accelerate synaptic elimination (Brown, 2021). Meanwhile, mitochondrial dysfunction triggers metabolic reprogramming and releases large amounts of DAMPs, which then activate multiple inflammatory cascades (Gülke et al., 2018).

### 3.3 Z-axis: functional outputs

The third dimension of the MNCC model encompasses the functional outputs generated by microglia-neuron communication, whose biological effects are inherently dual and context-dependent. Spatial heterogeneity serves as a key modifier. It reshapes the final outcomes of the same crosstalk events in different brain regions, tightly coupling the Z-axis functional spectrum to regional tissue microenvironments. Within the acutely affected infarct core, unrestrained microglial pro-inflammatory signaling triggers excitotoxicity, oxidative stress, and secondary neuronal necrosis. By contrast, moderately regulated microglial responses in the ischemic penumbra restrict lesion expansion, clear toxic debris, and launch early tissue repair cascades (Li et al., 2023). Phagocytic activity similarly exhibits a double-edged nature. In the infarct core, the efficient clearance of necrotic debris and myelin waste is highly beneficial, as it alleviates DAMP accumulation and prevents secondary injury. However, in peri-infarct areas, excessive or nonselective phagocytosis removes stressed but viable neurons and erodes intact synapses, thereby aggravating permanent neurological impairment (Chen et al., 2022).

Due to time- and space-related functional differences, the MNCC model avoids simple labels for microglial phenotypes. All signaling molecules and cell interactions lie on a continuous functional spectrum. One end of the Z-axis covers harmful outcomes, including expanded injury, severe inflammation, pathological phagocytosis and synapse loss. The other end covers beneficial outcomes, including reduced inflammation, debris clearance, synaptic remodeling and neural circuit repair. The same molecular pathway can produce opposite effects along the Z-axis. The MNCC model provides a comprehensive conceptual framework for reorganizing therapeutic strategies after ischemic stroke. The model emphasizes that these interactions are temporally evolving, spatially heterogeneous, communication-mode dependent, and functionally plastic. Consequently, effective interventions must move away from global microglial inhibition or broad immunosuppression. Instead, they must precisely reshape maladaptive communication modes while preserving or enhancing protective and reparative signaling pathways.

## 4 Therapeutic targeting through the lens of the MNCC model

### 4.1 Modulating inflammatory mediators

Interventions targeting inflammatory signaling cascades remain a core therapeutic direction in stroke research. Key druggable axes include the NLRP3 inflammasome, IL-1β maturation, HMGB1-TLR4 signaling, NF-κB transcriptional cascades, JAK/STAT pathway, and the cGAS-STING pathway (Panbhare et al., 2024; Bellut et al., 2023; Gou et al., 2020; Ri et al., 2023; Ji et al., 2026). Timely blockade of overactive acute inflammation can significantly alleviate secondary neurotoxicity. However, inflammatory mediators exert dual, phase-dependent roles in stroke. Beyond their acute toxicity, these molecules are dynamically involved in clearing necrotic tissue, stimulating angiogenesis, remodeling synapses, and facilitating long-term repair (Monsour et al., 2023; Xu et al., 2024). Sustained or complete inhibition of inflammatory signaling across all disease phases inevitably hinders long-term neurological recovery. Accordingly, anti-inflammatory strategies must be tailored to the stroke timeline: transiently suppressing excessive inflammation during the acute phase reduces secondary neuronal loss, whereas selected repair-related immune signals must be preserved or moderately activated during the subacute and chronic phases to support tissue regeneration.

### 4.2 Restoring homeostatic checkpoint signaling

Reconstruction of neuron-derived inhibitory immune checkpoint signaling represents a promising strategy to constrain pathological microglial overactivation after cerebral ischemia. Key checkpoints include the CX3CL1-CX3CR1, CD200-CD200R, and CD47-SIRPα axes. However, their therapeutic efficacy is strictly dependent on the stroke stage. In the acute phase, inhibiting CX3CR1-mediated microglial activation or deficiency in CX3CR1 alleviates acute neuroinflammation and exerts robust neuroprotection against cerebral ischemic injury (Tang et al., 2014). Conversely, during the subacute and chronic repair phases, moderate activation of this axis is required to regulate beneficial microglial phagocytosis, synaptic remodeling, and neural repair (Ge et al., 2022). Furthermore, therapeutic manipulation of immune checkpoints carries notable translational hurdles. Exogenous supplementation of CD200 or its mimetic peptides, the use of agonists to enhance CD200R signaling, or genetic upregulation of this pathway can effectively attenuate post-ischemic injury (Zhao et al., 2020; Sun et al., 2020). However, because the CD200-CD200R pathway is also widely expressed on peripheral immune cells and shares overlapping functions with other systemic immune pathways, isolated interventions often yield limited or inconsistent clinical efficacy (Ngwa et al., 2026).

### 4.3 Regulation of phagocytosis

Therapeutic tuning of microglial phagocytosis should serve a dual purpose: promoting the clearance of necrotic debris and myelin, while inhibiting phagoptosis of vulnerable neurons and excessive synapse elimination. Major candidate molecular targets include the phagocytic receptors TREM2, MerTK, and Axl, complement cascade components, microglial CR3, and “eat-me” recognition systems (Yu et al., 2025; Kaur et al., 2025; Brown, 2021). Within the infarct core, augmented phagocytic capacity accelerates the removal of cellular waste and facilitates the timely resolution of neuroinflammation. For instance, enhancing MerTK/Axl-mediated microglial efferocytosis promotes the clean clearance of apoptotic cells without amplifying pro-inflammatory cytokine release (Neher et al., 2013). Similarly, the intracerebroventricular injection of TREM2 agonistic antibodies or the intranasal delivery of TREM2-activating peptides enhances microglial recognition and clearance of damaged synapses and debris (Zhou Y. et al., 2025). By contrast, unrestrained phagocytic activity within the metabolically vulnerable penumbra eliminates functionally salvageable neurons and erodes intact synapses, worsening permanent neurological deficits. To counteract this, complement inhibitors such as anti-C1q antibodies or CR2-Crry fusion proteins can significantly reduce abnormal complement deposition in the peri-infarct region, thereby suppressing microglial over-phagocytosis of functional synapses (Sun et al., 2024; Alawieh et al., 2015]. This regional dichotomy underscores the critical need for spatially selective phagocytosis regulators. Developing therapeutic agents capable of discriminating irreversibly necrotic core cells from transiently stressed penumbral neurons would constitute a major breakthrough in stroke immunotherapy.

### 4.4 Engineering extracellular vesicle-based therapies

EVs constitute a highly promising delivery platform to shuttle protective microRNAs, functional proteins, and metabolic cargoes directly to injured ischemic tissues. Customized, bioengineered EVs can be rationally designed to suppress pro-inflammatory cascades, promote neuronal survival, stimulate angiogenesis, and facilitate synaptic reconstruction. Preclinical ischemic stroke models have validated the robust therapeutic potential of EVs isolated from mesenchymal stromal cells, neural stem cells, and genetically modified immune cells (Barbati et al., 2026; Xu et al., 2026; Nakazaki et al., 2026).

While these preclinical outcomes are encouraging, multiple bottlenecks hinder clinical translation. These include heterogeneous EV cargo compositions, challenges in large-scale standardized manufacturing, inconsistent cargo loading efficiencies, insufficient brain-targeting specificity, unpredictable *in vivo* biodistribution profiles, and unresolved long-term safety concerns (Herrmann et al., 2021; Luisotti et al., 2025). Furthermore, EV bioactivity varies drastically depending on the parent cell origin and the stroke stage. The rational development of EV therapeutics must therefore fully incorporate the temporal, spatial, and functional contexts defined by the MNCC model.

### 4.5 Targeting metabolic coupling

Metabolic interactions determine the intrinsic functional status of both neurons and microglia, fundamentally shaping the switch between neurotoxic and neuroprotective microglial phenotypes. Metabolic regulation remodels the immune phenotype of microglia, enhances neuronal stress tolerance, and alters intercellular signaling, thereby interrupting the vicious cycle of metabolic disorder, neuroinflammation, and neuronal injury at its upstream source. This regulatory process engages several core metabolic pathways, including AMPK signaling, SIRT family cascades, and pathways controlling mitochondrial quality, glucose metabolism, and lipid homeostasis (Lv et al., 2025; Zhang et al., 2026).

Despite encouraging efficacy in preclinical models, multiple barriers remain. The most critical issue is the double-edged nature of microglial metabolic flexibility. For instance, accelerated glycolysis is not only the metabolic basis for the pro-inflammatory polarization of microglia but also an essential energy source for their basic survival under ischemic conditions (Zhang et al., 2024). Forcibly inhibiting glycolysis depletes microglial energy, leading to cell death and the release of additional damage signals that exacerbate neuronal injury. A more favorable approach is to restore microglial metabolic flexibilitym, the ability to adaptively switch between oxidative phosphorylation and glycolysis (Zhang et al., 2026). According to the core tenets of the MNCC model, future metabolic therapeutic strategies should abandon generalized metabolic regulation in favor of precise interventions targeting stage-specific and region-specific metabolic coupling disorders.

### 4.6 Strategies targeting organelle transfer

Pharmacological and biological manipulation of intercellular organelle transfer and mitochondrial quality control represents a cutting-edge frontier in ischemic stroke management. The utilization of tunneling nanotubes (TNTs) to direct healthy mitochondria from supporting microglia to energetically compromised neurons offers an innovative strategy to directly rescue the ischemic penumbra (Scheiblich et al., 2024). Endogenous enhancement of this system can be achieved via agents such as melatonin, which upregulate TNT-mediated mitochondrial traffic and significantly alleviate ischemic injury (Yip et al., 2021). For exogenous therapeutic supplementation, the transplantation of mesenchymal stem cells or the direct microinjection of isolated, healthy mitochondria enables direct uptake by damaged neurons through TNT or EVs pathways (Liao et al., 2024; Sun et al., 2024). Theoretically, these approaches resolve acute neuronal bioenergetic failure while suppressing sterile neuroinflammation triggered by damaged endogenous organelles. However, this field remains largely preclinical with limited direct *in vivo* evidence from ischemic stroke models. Because uncontrolled bidirectional organelle exchange may trigger unforeseen pathological consequences, systematic research is required before clinical translation (Table 2).

**TABLE 2:** Therapeutic approaches targeting microglia-neuron crosstalk in ischemic stroke.

Therapeutic strategy category	Representative methods/Targets	Main mechanisms	Translational challenges	References
Modulating inflammatory mediators	NLRP3, IL-1β, HMGB1-TLR4, NF-κB, JAK/STAT, cGAS-STING pathways	Suppresses the production of inflammatory mediators to prevent nerve damage; avoid completely suppressing inflammation to preserve repair functions	Dual, phase-dependent roles of cytokines; sustained or complete inhibition may severely impair subacute/chronic tissue repair	Panbhare et al., 2024; Bellut et al., 2023; Gou et al., 2020; Ri et al., 2023; Ji et al., 2026; Monsour et al., 2023; Xu et al., 2024
Restoring homeostatic checkpoint signaling	CX3CL1-CX3CR1, CD200-CD200R “don’t-eat-me”signaling	Attenuates post-ischemic hyperactivation in the acute phase; promotes homeostatic surveillance, synaptic remodeling, and efferocytosis later	Narrow therapeutic window; extensive crossover with peripheral immune cells; risk of anemia and age-dependent variability	Pawelec et al., 2020; Zhao et al., 2019; Tang et al., 2014; He et al., 2025; Ge et al., 2022; Zhao et al., 2020; Sun et al., 2020; Ngwa et al., 2026
Precision regulation of phagocytosis and synaptic remodeling	Complement (C1q/CR2-Crry), PS masking (Annexin A5), Siglec-11, MerTK/Axl, TREM2 agonists	Inhibit aberrant synaptic phagocytosis, promote efferocytosis, induce reparative phenotype	Poor spatiotemporal precision; difficulty in discriminating core debris from salvageable penumbral elements	Yu et al., 2025; Kaur et al., 2025; Brown, 2021; Neher et al., 2013; Zhou T. et al., 2025; Sun et al., 2024; Alawieh et al., 2015
EV-based modulation	Mesenchymal stromal cell-derived EVs, engineered EVs	Shuttles protective small non-coding RNAs, lipids, and proteins across the BBB; induces an anti-inflammatory phenotype; promotes neurogenesis	Heterogeneity; lack of standardized large-scale manufacturing; unpredictable *in vivo* biodistribution; stability concerns	Barbati et al., 2026; Xu et al., 2026; Herrmann et al., 2021; Luisotti et al., 2025
Targeting metabolic coupling and mitochondrial communication	AMPK signaling, SIRT family cascades, mitochondrial quality control, glucose/lipid homeostasis regulation	Restores microglial metabolic flexibility	Forced or generalized glycolysis inhibition may deplete microglial energy and trigger cell death, worsening microenvironmental distress	Lv et al., 2025; Zhang et al., 2026; Wang Q. et al., 2025; Wei et al., 2024; Zhang et al., 2024
Strategies targeting organelle transfer	Melatonin-induced TNT upregulation, transplantation of MSCs or isolated healthy mitochondria via TNT/EV pathways	Rescues neuronal bioenergetic failure; directly transfers functional donor mitochondria from microglia to energetically compromised neurons	Predominantly preclinical; lacks solid *in vivo* validation; long-term functional effects and potential immunogenicity remain unclear	Scheiblich et al., 2024; Piao et al., 2026; Yip et al., 2021; Liao et al., 2024; Sun et al., 2024

## 5 Challenges and future directions

### 5.1 Microglial heterogeneity

Microglia exhibit profound heterogeneity across different brain regions, disease stages, age groups, sexes, and pathological comorbidities. Single-cell transcriptomic studies have revealed a wide range of distinct functional states in microglia. These states go far beyond the traditional M1/M2 polarization framework. Future research should focus on defining specific molecular signatures. Such signatures would correspond to discrete states along the MNCC model. Furthermore, clarifying the exact evolutionary translation of these distinct microglial subsets into human stroke pathology represents an indispensable prerequisite for the identification of clinical targets.

### 5.2 Shifting therapeutic windows

A single signaling pathway can exert opposing effects during stroke progression. It may confer beneficial functions at one time point, yet induce pathological damage at another. Consequently, therapeutic windows for stroke interventions cannot be calculated purely as a function of chronological time elapsed from symptom onset. Instead, they must be dynamically derived from real-time parameters, including tissue viability, reperfusion efficacy, neuronal metabolic status, and the temporal trajectory of neuroinflammation. Identifying and validating reliable peripheral biomarkers capable of capturing these fluid MNCC states in real time remains an urgent clinical priority.

### 5.3 Spatial heterogeneity and regional targeting

Distinct microglia-neuron communication landscapes exist across the ischemic core, the penumbra, the peri-infarct cortex, and distal non-ischemic structures (Dou et al., 2023). Therapeutic interventions may produce protective effects in the ischemic core. However, they can also lead to adverse outcomes in the penumbral region. This contrast highlights the critical impact of spatial heterogeneity on treatment efficacy. Future therapeutic strategies should integrate multiple approaches, including spatial omics technology, high-resolution advanced imaging, and site-specific targeted delivery systems. The overall goal is to achieve precise regional.

### 5.4 Biomarker limitations

Current clinically available biomarkers fail to comprehensively and accurately reflect the dynamic MNCC states in stroke patients. Systemic cytokine profiling, conventional neuroinflammatory neuroimaging, and circulating EV cargo characterization offer only fragmented, static snapshots of central microenvironment. None of these single indicators is sufficient to precisely locate a patient’s pathological status within the MNCC continuum. Developing novel, multimodal biomarker matrices that merge real-time neuroimaging signatures, blood-based molecular panels, and computational machine-learning algorithms will be essential to accurately track MNCC dynamics *in vivo*.

### 5.5 Need for clinically compatible intervention strategies

The overwhelming majority of preclinical stroke studies are conducted utilizing young, healthy laboratory animals, which fail to capture the clinical complexity of human stroke. The typical stroke patient is an elderly individual burdened with multiple chronic comorbidities, such as hypertension, diabetes, hyperlipidemia, and age-dependent systemic inflammation. These clinical confounding factors profoundly alter baseline microglial priming and amplify neuronal vulnerability to ischemic death. Future preclinical research should adopt more clinically relevant animal models. Furthermore, the dynamic shifts in BBB permeability post-stroke, unsatisfactory drug delivery routes, and the risks of off-target systemic immunosuppression remain critical operational bottlenecks that must be resolved to ensure successful clinical translation.

## 6 Conclusion

In this Review, we propose the MNCC model as a unifying conceptual framework that moves beyond binary harmful-versus-protective classifications of post-stroke microglial responses. By mapping microglia-neuron crosstalk along temporal, communication-mode, and functional-output axes, the MNCC model reconciles historically discordant findings: the same molecular pathway can amplify acute injury yet support late repair, depending on timing, concentration, and anatomical context. This continuum perspective offers a more physiologically faithful interpretation of the evolving post-ischemic microenvironment than stage-based or binary models.

Looking forward, critical knowledge gaps remain. Future work must define definitive molecular signatures that map specific microglial communication states onto positions within the MNCC, and develop non-invasive biomarkers capable of tracking these dynamics *in vivo*. Advances in single-cell transcriptomics, spatial omics, longitudinal intravital imaging, and clinically relevant animal models will be essential to refine this framework. Ultimately, the MNCC model provides a strategic foundation for transforming mechanistic complexity into time- and region-specific therapeutic precision, shifting the goal from global microglial suppression to targeted restoration of balanced cellular dialogue.
